# Synthesis, Characterization, PXRD Studies, Theoretical Calculation, and Antitumor Potency Studies of a Novel N,O-Multidentate Chelating Ligand and Its Zr(IV), V(IV), Ru(III), and Cd(II) Complexes

**DOI:** 10.1155/2022/2006451

**Published:** 2022-06-28

**Authors:** Hussein H. Elganzory, Safaa S. Hassan, Samar A. Aly, Ehab M. Abdalla

**Affiliations:** ^1^Department of Chemistry, College of Science, Qassim University, Buraidah 51452, Saudi Arabia; ^2^Chemistry Department, Faculty of Science, Cairo University, Giza 12613, Egypt; ^3^Department of Environmental Biotechnology, Genetic Engineering and Biotechnology Research Institute, University of Sadat City, Sadat 32958, Egypt; ^4^Chemistry Department, Faculty of Science, New Valley University, Alkharga 72511, Egypt

## Abstract

A new series of Zr(IV), V(IV), Ru(III), and Cd(II) complexes with the ligand N-((5-hydroxy-4-oxo-4H-pyran-3-yl)methylene)-2-(p-tolylamino)acetohydrazide (*H*_*2*_*L*) have been prepared. FT-IR, ^1^H-NMR, electronic spectra, powder X-ray, and thermal behavior methods were applied to elucidate the structural composition of new compounds. Geometry optimization for all synthesized compounds was conducted using the Gaussian09 program via the DFT method, to obtain optimal structures and essential parameters. Moreover, the antibacterial and antitumor activity of the ligand and its complexes were studied, where the Cd(II) complex acquires probably the best antibacterial activity followed by the Ru(III) complex towards bacterial species than others when using ampicillin and gentamicin were used as standard drugs. The complexes exhibited interestingly antitumor potential against the MCF-7 breast cancer cell line. The cytotoxicity of the new complexes has been arranged to follow the order: Ru(III) complex > Cd(II) complex > Zr(IV) complex > V(IV) complex > ligand. Molecular docking was performed on the active site of ribosyltransferase and obtained good results. Structure-based molecular docking is used to identify a potential therapeutic inhibitor for NUDT5.

## 1. Introduction

The derivatives of hydrazone constitute a significant class of compounds that have been used in various therapeutic chemistry applications [1]. These applications are significant because of the wide range of their pharmacokinetic properties [2–5], especially their importance in drug detection programs [6, 7]. Numerous studies have confirmed that the hydrazone and carbaldehyde derivatives and their complexes have a wide series of biological properties [8–11], such as anticancer [12–15], antibacterial [16–18], antimicrobial [19–21], antifungal [22, 23], antimalarial [24], antiviral [25], antimycobacterial [12, 26], antileishmanial [25, 27], antiplatelet [28], antianalgesic, antitubercular, anticonvulsant [29], antiuropathogenic [30], antiproliferative [31], antiarthritic [32], and antioxidant [33–35] properties and are potent immunomodulatory agents [36] and antiangiogenic agents in atherosclerosis [33]. Moreover, they play an important role in treating Alzheimer's disease [37, 38]. Recently, a new ligand of ((Z)-2-(phenylamino)-N'-(thiophen-2-ylmethylene) acetohydrazide) [39] was used to synthesis Pd(II), Cd(II), Cu(II), and Cu(I) complexes, where the ligand behaves as a neutral bidentate or tetradentate, the Pd(II) complex is square planar, and Cu(II) and Cu(I) complexes are square pyramidal in geometry. However, the Cd(II) complex is tetrahedral in geometry. The elemental analyses display that complexes of (Pd(II) and Cd(II) have 1L : 1M stoichiometry, while Cu(II) and Cu(I) have 2L : 1M and 3L : 2M stoichiometry, and the biological applications such as antibacterial activity and antioxidant of the ligand and its complexes are reported [39]. Also, many ligands such as N´-[(E)-(6-fluoro-2-hydroxyquinoline-3-yl)methylidene]pyridine-3-carbohydrazide, N´-[(E) -(6-fluoro-2-hydroxyquinoline-3-yl)methylidene]pyridine-4-carbohydrazide, N´-[(E)-(6-fluoro-2-hydroxyquinoline-3-yl)methylidene]-6-methylpyridine-3-carbohydrazide, 2-[(7-bromo-2,3-dihydro-1H-inden-4-yl)oxy]-N´-[(E)-(6-fluoro-2-hydroxyquinoline-3-yl) methylidene]acetohydrazide, and 2-(2,3-dihydro-1H-inden-4-yloxy)-N´-[(E)-(6-fluoro-2-hydroxyquinoline-3-yl)methylidene] acetohydrazide [40] were used to prepare Cu(II) and Zn(II) complexes, where the experimental and theoretical values were calculated for 1 : 2 ratio of metal: ligand stoichiometry; all complexes have an octahedral geometry, and most of the complexes displayed 100% inhibitory activity against *Mycobacterium tuberculosis*.

From the previous work and the wide applications of acetohydrazide and carbaldehyde derivatives, the ligand N'-((5-hydroxy-4-oxo-4H-pyran-3-yl)methylene)-2-(p-tolylamino)acetohydrazide was synthesized, which is considered a modification of a previously used ligand and used as a chelator of Ru(III), Cd(II), Zr(IV), and V(IV) where the characterization, theoretical calculation, and the effect of these compounds on the bacterial species and MCF7 cancer cell lines were studied.

The ruthenium(III) complexes have been synthesized for their eclectic cytotoxic effects in vitro and hopeful anticancer properties in vivo, leading to a few candidates in developed clinical experiments aiming at treating the stability, solubility, and cellular uptake issues of depressed molecular weight Ru(III)-based components [41, 42]. Cd(II) complexes with Schiff base ligand can be used as an active emitting layer and exhibit photophysical properties [43] and showed anticancer activities against human liver cancer HepG2 cell line and human colon cancer HCT116 cell line [44]. Moreover, they showed excellent antibacterial activity against different bacterial strains [45, 46]. The Zr(IV) cation with its relatively small ionic radius and high positive charge number shows the characteristics of a hard Lewis acid, and it is perfect for strong complexation [47]. Zr(IV) complexes have a metalorganic framework that can be used for a variety of catalytic processes, industrial purposes, and pharmaceutical applications [44, 48, 49]. V(IV) complexes are reported to show various biological characteristics including antitumor, antimicrobial, antiobesity, antihyperlipidemic, and antihypertension activities, insulin-enhancing action, and improvement of oxygen-carrying efficiency of hemoglobin and myoglobin [50–53]. Vanadium complexes are also used for lowering of blood glucose [54–56] and natriuretic and diuretic effects.

Hence, the present work aims to study the preparation and spectroscopic characterization of Zr(IV), V(IV), Ru(III), and Cd(II) complexes of the ligand N'-((5-hydroxy-4-oxo-4H-pyran-3-yl)methylene)-2-(p-tolylamino)acetohydrazide and study the antibacterial and antitumor activity of the ligand and its complexes.

## 2. Experimental

### 2.1. Reagents

All chemicals such as 2-(p-tolylamino)acetohydrazide, 5-hydroxy-4-oxo-4H-pyran-3-carbaldehyde, ZrOCl_2_, VOSO_4,_ RuCl_3_, and CdCl_2_ were purchased from Sigma-Aldrich Chemie (Germany) and Fluka. Organic solvents, e.g., absolute ethanol, were of commercially available reagent grade and used without purification.

### 2.2. Synthesis of the Hydrazone Ligand

In a round flask, 1.792 g of 2-(p-tolylamino)acetohydrazide (0.01 mole) and 1.40 g of 5-hydroxy-4-oxo-4H-pyran-3-carbaldehyde (0.01 mole) were mixed in 20 ml of absolute ethanol. The resulting mixture was blended at room temperature for about 6 h [1]. The resulting precipitate was filtered off, washed several times with ethanol and diethyl ether, and then dried in a vacuum.

C_15_H_15_N_3_O_4_ (H_2_L) : yellow, M.W: 301.3, Yield = 94%, anal. calc: C, 59.80; H, 5.02; N, 13.95. Found (%): C, 59.76; H, 4.98; N, 13.92. FTIR (KBr, cm^−1^): 3392 (OH/H_2_O), 3202 (N-H), 1677 (C = O)_side_, 1639 (C = O)_ring_, and 1604 (C = N). Electronic spectra in DMF solution: *λ*_max_: 341, 399. ^1^HNMR (DMSO-d6): *δ* (ppm) = 2.10 (s, 3H, CH_3_), 3.91 (s, 2H, NCH_2_), 5.94 (s, 1H, NH), 6.57 (s, 1H, NCH), 6.70–6.75 (m, 4H, Ph-H), 7.11 (s, 2H, pyran-H), 11.10 (bs, 1H, NHC = O), 15.90 (bs, 1H, OH); ^13^CNMR: *δ* (ppm) = 19.2 (CH_3_), 45.3 (NCH_2_), 154.7 (C = N), 116.8, 128.0, 163.1, 179.1, 179.5 (pyran-C), 113.4, 113.4, 126.8, 129.8, 129.8, 144.6 (Ph-C), 173.5 (C = O); C_15_H_14_N_3_O_4_ (300.0); calc.: C, 58.7; H, 4.2; N, 14.7; found: C, 58.2; H, 4.5, N, 15.2.

### 2.3. Synthesis of Metal Complexes

**Scheme 1:**
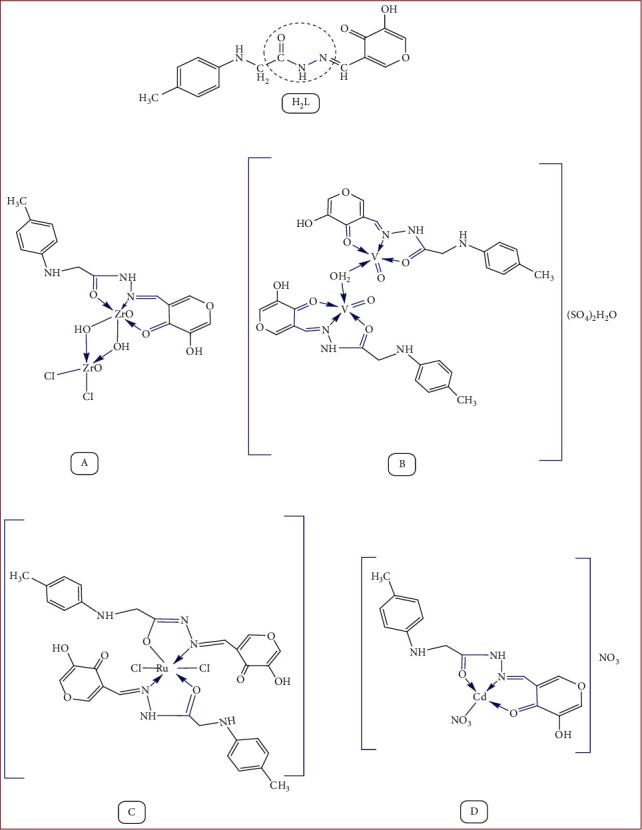
The suggested chemical structures of the ligand and its metal complexes.

In the boiling flask, a stoichiometric amount of the appropriate metal salt (1 mmol; 0.178 g Zr(IV); 0.163 g of V(IV); 0.207 g of Ru(III); and 0.174 g of Cd(II)) was added to 0.301 of the ligand (1 mmol) in the same solvent EtOH (20 ml) in accordance with a general procedure (Scheme 1). The reaction mixture was then refluxed at 60°C and stirred for 6 hr. The resulting product was filtered off from the mixture, thoroughly washed with ethanol to remove any traces of unreacted starting materials, and then dried in a vacuum [39]. The purity of the complexes was checked by TLC.

The yields and characterization details for the complexes are presented as follows.

#### 2.3.1. Zr(IV) Complex

Yellow, M.W: 620.66, [C_15_H_17_Cl_2_N_3_O_8_Zr_2_], yield = 88%, anal. calc: C, 29.03; H, 2.76; Cl, 11.42; N, 6.77; Zr, 29.40. Found (%): C, 28.97; H, 2.71; Cl, 11.39; N, 6.72; Zr, 29.33. FTIR (KBr, cm^−1^): 3399 (OH/H_2_O), 3100 (N-H), 1691 (C = O)_side_, 1644 (C = O)_ring_, 1582 (C = N), 571 (M-O), 506 (M-N). Electronic spectra in DMF solution: *λ*_max_: 338 and 479 nm.

#### 2.3.2. V(IV) Complex

Yellow, M.W: 964.63, [C_30_H_34_N_6_O_20_S_2_V_2_], yield = 83%, anal. calc: C, 37.35; H, 3.55; N, 8.71; V, 10.56. Found (%): C, 37.12; H, 3.49; N, 7.68; V, 10.52. FTIR (KBr, cm^−1^): 3399 (OH/H_2_O), 3100 (N-H), 1691 (C = O)_side_, 1644 (C = O)_ring_, 1582 (C = N), 506 (M-O), 571 (M-N). Electronic spectra in DMF solution: *λ*_max_: 338 and 479 nm.

#### 2.3.3. Ru(III) Complex

Deep brown, M.W: 773.6, [C_30_H_29_Cl_2_N_6_O_8_Ru], yield = 86%, anal. calc: C, 46.58; H, 3.78; Cl, 9.17; N, 10.86; Ru, 13.07. Found (%): C, 46.53; H, 3.74; Cl, 9.11; N, 10.82; Ru, 13.02. FTIR (KBr, cm^−1^): 3433 (OH/H_2_O), 2925 (N-H), 1689 (C = O)_side_, 1650 (C = O)_ring_, 1553 (C = N), 605 (M-O), 560 (M-N). Electronic spectra in DMF solution: *λ*_max_: 340 and 519 nm.

#### 2.3.4. Cd(II) Complex

Yellow, M.W: 537.7, [C_15_H_15_CdN_5_O_10_], yield = 89%, anal. calc: C, 33.51; H, 2.81; N, 6.77; Cd, 20.91. Found (%): C, 33.24; H, 2.76; N, 6.62; Cd, 20.89. FTIR (KBr, cm^−1^): 3422, 3327 (OH/H_2_O), 3234, 3104 (N-H), 1669 (C = O)_side_, 1638 (C = O)_ring_, 1603 (C = N), 587 (M-O), 455 (M-N). Electronic spectra in DMF solution: *λ*_max_: 338 and 378 nm.

### 2.4. Physical Measurements

The Fourier transform infrared (FT-IR) spectrum was measured (4000–400 cm^−1^) in KBr discs using Nenexeus-Nicolidite-640-MSA FT-IR, Thermo-Electronics Co. In the DMF solution, the UV-visible absorption spectra were measured using a 4802 UV-Vis double-beam spectrophotometer. The ^1^H-NMR spectra have been recorded in DMSO-d6 as a solvent using a Varian Gemini 200 NMR spectrophotometer and Varian-Oxford Mercury at 300 MHz, respectively. Thermal analysis (TG/DTG) was obtained using a Shimadzu DTA/TG-50 Thermal Analyzer with a heating rate of 10°C/min in a nitrogen atmosphere with the rate of 20 mL/min using platinum crucibles in the range of ambient temperature up to 800°C. Mass spectra were acquired by the electron impact (EI) ionization technique at 70 eV on a Hewlett–Packard MS-5988 GC-MS instrument at the Microanalytical Center, National Research Center, Egypt. X-ray powder diffraction analyses of solid samples were measured using a APD 2000 PROModel GNR-X-ray diffractometer (NRC, Tanta University, Egypt). X-ray diffractometer is ready with Cu K*α* radiation (*λ* = 1.540 56 Å). Most powder diffractometers use Bragg–Brentano parafocusing geometry. The X-ray tube applied was a copper tube operating at 40 KV and 30 mA. The scanning range (2*θ*) was 5°–90° with a step size of 0.050° and a counting time of 2 s/step. Quartz was utilized as the standard material, accurate for the instrumental extension. This identification of the complexes was done by a known method from the fit identified Scherer formula, and the average crystallite size (*D*) is

(1) $$\begin{array}{c} D=\left(\frac{\mathrm{K\lambda}}{\mathrm{\beta cos\theta}}\right), \end{array}$$

where *λ* is the X-ray wavelength in nanometers, *K* is a factor related to crystallite shape, with a value of about 0.9, and *β* is the peak width at half-maximum height. The value of *β* in the 2*θ* pivot of diffraction shape should be in radians. *θ* is the Bragg angle and can be in radians since the Cos*θ* is suitable with the same number.

### 2.5. Kinetic and Thermodynamic Parameters for the Complexes

The kinetic and thermodynamic parameters of the decomposition stages of the complexes (**C, D**) were determined from the TGA thermogram using the Coats–Redfern equation [57]. The values of the activation energy *E∗*, Arrhenius constant *A*, activation entropy *S∗*, activation enthalpy *H∗*, and free energy of activation *G∗* are calculated by applying Coats–Redfern equation for *n* = 1.

(2) $$\begin{array}{c} \log\left[\frac{-\log\left(1-x\right)}{T^{2}}\right]=\log\frac{AR}{\theta E^{*}}\left(1-\frac{2RT}{E^{*}}\right)-\frac{E^{*}}{2.303RT}, \end{array}$$

where *x* is the fraction decomposed, *R* is the gas constant, and *θ* is the heating rate. Since (*1-2RT/E∗*) ≃ 1, a plot of the left-hand side of equation (2) against *1/T* gives a straight line from its slope and intercept, and ***E****∗* and ***A*** were calculated. The entropy of activation ***S****∗*, enthalpy of activation ***H****∗*, and the free energy change of activation ***G****∗* were calculated using the following equations:

(3) $$\begin{array}{c} S^{*}=R\left[1\text{n}\left(\frac{Ah}{kT}\right)\right]R, \end{array}$$

(4) $$\begin{array}{c} H^{*}=E^{*}-RT, \end{array}$$

(5) $$\begin{array}{c} G^{*}=H^{*}-TS^{*}, \end{array}$$

where *k* is Boltzmann's constants and *h* is Planck's constants.

### 2.6. Quantum Chemical Calculation (QCC)

The input files of all compounds were prepared with GaussView 5.0.8 [58]. Gaussian 09 rev. A.02 [59] software was used for all calculations by the DFT/B3LYP method. 6/31G and LANL2DZ are the standard basis sets for the synthesized ligands and their metal complexes. All docking steps were done using MOE 2008 (Molecular Operating Environment) software to simulate the binding model of these compounds into ATP binding sites of 3GEY transferase and the NUDT5 proteins. The protein crystal structures were obtained from the Protein Data Bank (PDB).

### 2.7. Antibacterial Assay

The antimicrobial activity of synthesized compounds was determined by the agar well diffusion method [60]. All the compounds were tested in vitro for their antibacterial activity against *Staphylococcus aureus (ATCC:13565)* and *Streptococcus mutans (ATCC:25175)* (Gram-positive bacteria) and *Escherichia coli (ATCC:10536)* and *Klebsiella pneumonia (ATCC:10031)* (Gram-negative bacteria) using nutrient agar medium. Ampicillin and gentamicin were utilized as standard medications for Gram-positive and Gram-negative bacteria. DMSO was used as a control solvent.

### 2.8. MTT Assay for Anticancer Activity

The cytotoxicity of the synthesized ligand and complexes against the MCF-7 breast cancer cell line was examined by MTT assay (MTT: 3(4,5-dimethylthiazol-2-yl)-2,5-diphenyltetrazolium bromide). The cell suspension was diluted with a complete medium to a concentration of 5 × 10^4^ cell·mL^−1^. The cell suspension (100 *μ*L) was pipetted into each well of a 96-well plate (about 5000 cells per well). The 96-well tissue culture plate was inoculated with 1 × 10^5^ cells/ml (100 *μ*L/well) and incubated at 37°C for 24 hours to develop a complete monolayer sheet. The growth intermediate was poured from 96-well microtiter plates after a confluent sheet of cells was created, and the cell monolayer was washed twice with washing media. Two-fold dilutions of the tested sample were made in RPMI medium with 2% serum (maintenance medium). 0.1 ml of each dilution was tested in various wells, leaving 3 wells as control, thus only receiving a maintenance medium. The plate was incubated at 37°C and then examined. Cells were scanned for any toxicity physical signs, e.g., partial or complete absence of the monolayer, rounding, retractability, or cell granulation.

The solution of MTT was prepared (5 mg/ml in PBS) (BIO BASIC CANADA INC). A 20 *μ*L MTT solution was added to each well and then placed on a vibration table, 150 rpm for 5 minutes, to completely combine the MTT into the media. Thereafter, it was incubated (37°C, 5% CO_2_) for 1–5 hours to allow the MTT to be metabolized. The medium was then dumped off. A dry plate was placed on paper towels to remove remains if necessary. Resuspended formazan (MTT metabolic product) was resuspended in 200 *μ*L DMSO and then placed on a vibration desk, at 150 rpm for 5 minutes, to completely combine the formazan into the solvent. The optical density was recorded at 560 nm, and the background at 620 nm was subtracted, since it is important to directly correlate optical density with cell quantity [61–63].

## 3. Results and Discussion

### 3.1. Physicochemical Properties

All metal chelates are colored and stable towards air and moisture. The analytical results for the complexes are consistent with the proposed molecular formulas and confirm the formation of 2 : 1 of Zr(IV), 2 : 2 of V(IV), 1 : 2 of Ru(III), and 1 : 1 (M : L) of Cd(II) complexes (Table 1). The molar conductance values for the complexes in 10^−3^ M DMF solution are in the range of 46–89 Ω^−1^cm^2^·mol^−1^ for Zr(IV), V(IV), Ru(III), and Cd(II) complexes. The values of the Zr(IV) and Ru(III) reveal their nonelectrolytic nature, while the values of the V(IV) and Cd(II) complexes reveal their electrolytic nature [64, 65].

### 3.2. ^1^H-NMR and ^13^C-NMR Spectra

The ^1^H-NMR spectrum of the ligand was verified in DMSO-d6. It exhibits one signal at d 5.94 ppm assigned to the NH proton and a broad single peak observed at 11.1 ppm assigned to the NHC = O proton. Furthermore, the spectrum displays multiple signals at (6.70–6.75 ppm) assigned to aromatic ring protons. Moreover, the spectrum depicts singlet signals (7.11 ppm) corresponding to pyrene protons. Thus, the ^1^H-NMR result supports the assigned geometry. The ^1^H-NMR spectrum of the ligand was verified in DMSO-d6 solution (d ppm) (Figure S1A). The ^1^H-NMR spectrum exhibited a signal at 2.36 (s, 2H, CH_2_), 4.14 (br, 1H, NH), 6.58–7.63 (m, 8H, aromatic system), 5.94 (s, 1H, CH), and 11.36 (s, 1H, NH) (amide a to hydrazone linkage).

The ^13^C-NMR spectrum of the ligand (DMSO-d6) (Figure S1B) features a signal at 19.2 ppm corresponding to the methyl group, while the methylene carbon was assigned at 45.3 ppm. The aromatic carbons of the phenyl ring are observed at 113.4, 126.8, 129.8, 129.8, and 144.6 ppm, whereas the pyrane ring carbons appeared at 116.8, 128.0, 163.1, 179.1, and 179.5 ppm. In addition, two carbon atoms appeared at 154.7 and 173.5 ppm that corresponded to (C = N) and (C = O), respectively.

### 3.3. FT-IR Spectra

**Figure 1:**
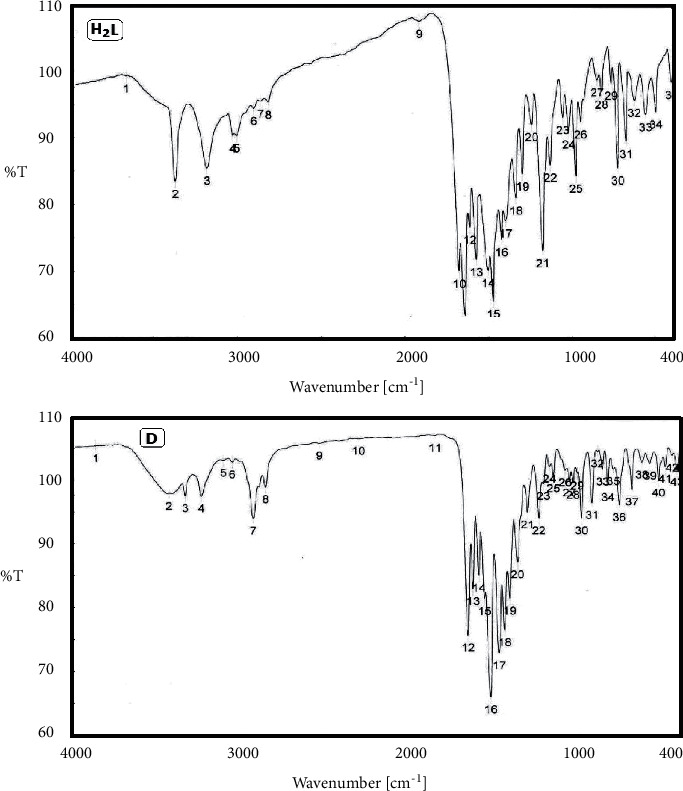
FT-IR spectra of the ligand and Cd(II) complex (D).

FT-IR spectra of the ligand and its metal complexes are depicted in Table 2, Figure 1, and Figure S2. The ligand spectrum shows a band at 1604 cm^−1^ which corresponds to the (−C = N) stretching vibration [66]. On complexation, this band is shifted to a lower frequency (1582, 1595, 1553, and 1603 cm^−1^) for Zr(IV), V(IV), Ru(III), and Cd(II) complexes, respectively.

The red shift is a proof that the azomethine nitrogen atoms get shared in complex formation. The IR spectrum of the ligand additionally showed a broad band at 3202 cm^−1^ due to the stretching vibration of the ***ν***(N-H) group. On complexation, the IR spectra of all complexes displayed that the bands of the imine groups have been shifted to a lower wave number (3100, 2625, 2925, and 3104 cm^−1^) for Zr(IV), V(IV), Ru(III), and Cd(II) complexes, respectively, than those of the free Schiff base ligand. The broad bands in the 3392 cm^−1^ region are due to the hydroxy group, and this band was shifted to higher frequencies (3399, 3434, 3433, and 3422 cm^−1^) for Zr(IV), V(IV), Ru(III), and Cd(II) complexes, respectively, which did not participate in the complex formation, where we note the appearance of new bands within the ranges 560–455 (*ν*M―N) and 621–571 (*ν*M―O) cm^−1^ and which confirms the participation of N atom of the azomethine group [67] and (carbonyl) O atom in formation of the complexes to form a hexagonal ring with the carbaldehyde moiety, where the bands at ranges (571, 506), (621, 553), (605, 560), and (587, 455) cm^−1^ corresponded to ***ν***(M―O) and ***ν***(M―N) for Zr(IV), V(IV), Ru(III), and Cd(II) metal complexes, respectively [39]. On the other hand, the broad band at 1677 and 1639 cm^−1^ was assigned to the (***ν***(C = O) side and ring) and shifted to 1692 and 1644 in the Zr(IV) complex, 1692 and 1644 in the Ru(III) complex, 1689 and 1650 in the V(IV) complex, and 1669 and 1638 cm^−1^ in the Cd(II) complex, confirming the participation of (***ν***(C = O) side and ring) in the complexation, which excludes the possibility of the participation of the hydroxyl group.

The presence of two strong bands at 1375 and 1315 cm^−1^ was assigned to ***ν***_**as**_(NO_3_) and ***ν***_**s**_(NO_3_), indicating monodentate nitrate groups [68, 69].

### 3.4. The Electronic Spectra and Magnetic Data

**Figure 2:**
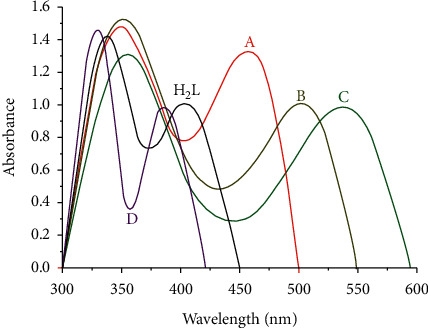
Electronic spectra of the synthesized ligand and its (a) Zr(IV), (b) V(IV), (c) Ru(III), and (d) Cd(II) complexes.

The UV-Vis spectra of the hydrazone ligand and its complexes of Zr(IV), V(IV), Ru(III), and Cd(II) were recorded in 10−3 DMF solution in the range of 200–800 nm at room temperature. The values of the maximum absorption wavelength (*λ*_max_) and magnetic moments (*μ*_eff_) are listed in Table 3, and the spectra are presented in Figure 2. The absorption spectrum of the ligand showed two absorption bands in the ultraviolet region [70]. The first high-intensity bands were observed at *λ*_max_ = 341 nm, and the second low-intensity bands were observed at *λ*_max_ = 399 nm. The two bands are attributed to the (n-*π∗*) transition associated with the azomethine group [60]. The band of the high wavelength side exhibited a bathochromic shift relative to its free ligand. The absorption bands of the first wavelength in the ligand slightly changed for metal complexes, while the second band strongly changed, where the electronic spectra of Zr(IV), V(IV), and Ru(III) complexes of this band were shifted to (338–479), (351–555), and (340–519) nm. The first band of complexes at 338, 351, and 340 is attributed to the (n-*π∗*) transition. However, the second band in the visible region at 479, 555, and 519 is considered to arise from (d–d) transitions. The electronic spectrum of the Cd(II) complex exhibited two bands at 338 and 378 nm, referring to (n-*π∗*) transition associated with the azomethine group. At room temperature, the magnetic moment values of the V(IV) complex is 1.6 ppm, according to spin-spin interaction between the V ion in the binuclear complex causing low value of the magnetic moment [53], Ru(III) complex is 1.74 B.M. which are close to the spin-only value of one unpaired electron. However, the Cd complex showed a diamagnetic character. These results indicated that the ligand coordinates to Zr(IV), V(IV), Ru(III), and Cd(II) are in accordance with the results of other spectral data. According to modern molecular orbital theory [71], any factors that can influence the electronic density of the conjugated system must result in the bathochromic or hypsochromic shift of absorption bands. Here, in the case of the metal complexes with the same ligand, the main reason for bathochromic shifts is generally related to the electronegativity of the different metal ions [72].

### 3.5. ESI-MS Spectra

The mass spectrum has been increasingly used to demonstrate the molecular structure of the ligand and complexes.  Figure S3 shows the mass spectrum of the ligand and its complexes of Zr(IV) and V(IV). The mass spectrum of the ligand gave a molecular ion peak at *m*/*z* = 302.21 (41%), which corresponds to C_15_H_15_N_3_O_4_ (calc. 301.3 amu) supporting the suggested structure. The molecular weights of various fragments of the ligand are consistent with the peaks of various intensities at *m*/*z* 271.81 (45%) corresponding to C_14_H_13_N_3_O_3_, 191.12 (21.73%) (calc. 194.01) to C_8_H_9_N_3_O_3_, 163.45 (56%) (calc. 165.04) to C_7_H_6_N_2_O_3_, 125.99 (39%) (calc. 123.11) to C_6_H_5_NO_2_, and 94.31 (12%) (calc. 96.09) to C_5_H_4_O_2_. On complexation, the mass spectra of Zr(IV) and V(IV) complexes display molecular ion peaks at *m*/*z* 621.11 (40%) and 965.62 (42%), and these data are in good agreement with the proposed molecular formulas for complexes (calc. 620.66 and 964.63 amu), respectively. The mass fragmentation pattern of the ligand and complexes are presented in Scheme S1, where the multipeak pattern of the mass spectra gives a series of peaks corresponding to the various fragments.

### 3.6. X-Ray Diffraction

**Figure 3:**
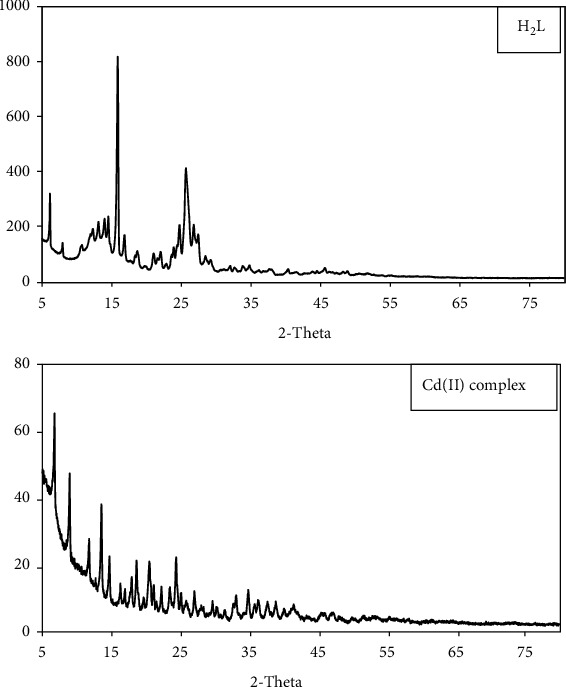
PXRD powder pattern of the ligand and Cd(II) complex.

Since the growth of single crystals of the synthesized complexes failed, PXRD was performed. The powder diffraction patterns of the ligand and its complexes of Zr(IV), Ru(III), and Cd(II) were recorded over 2*θ* = 5–80° (Table 4 and Figures 3, S4, and S5). The position of the highest intensity peak was determined, along with the width of this peak at half-maximum and the *d* spacing. The diffractogram of ligand displays a reflection with a maximum at 2*θ* = 15.822°, corresponding to a *d* value of 0.559119.

The patterns reveal well-defined crystalline peaks indicating the crystalline nature of the ligand and Cd complex, while Zr(IV) and Ru(III) complexes are amorphous in nature [60, 63]. The average particle sizes of the ligand and Cd complexes were calculated using the Scherer equation [66, 73], which were 37.48 (ligand) and 42.42 (Cd/ligand) nm.

### 3.7. Thermal Studies

**Figure 4:**
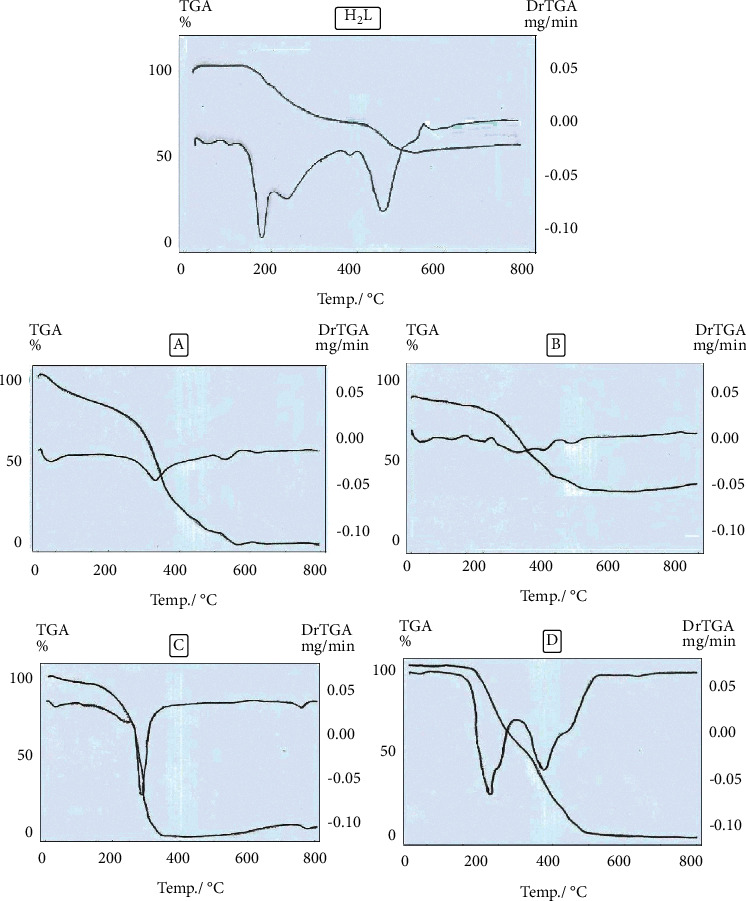
TGA/DTG curves of the synthesized ligand and its (a) Zr(IV), (b) V(IV), (c) Ru(III), and (d) Cd(II) complexes.

Thermogravimetric analysis (TGA) was carried out to probe the thermodynamic stability of the obtained compounds, as well as to collect information about the lattice guests. The TG and differential thermogravimetric (DTG) analyses for the ligand and Zr(IV), V(IV), Ru(III), and Cd(II) metal complexes over the 10°–800°C temperature range are shown in Table 5 and Figures 4(a)–4(e). The TG curves for the ligand showed two weight-loss events. The first decomposition was conducted at 197°–391°C and was accompanied by a weight loss of 60.31 (calc. 60.11)%, which is interpreted as the loss of C_7_H_5_N_2_O_4_ moiety. Over 391°C, the ligand decomposition was complete.

The TG curves for Zr(IV), V(IV), Ru(III), and Cd(II) complexes showed three weight-loss events. The first decomposition step occurred between 20° and 310°C, and it was accompanied by a weight loss of 18.90, 21.73, 8.98, and 36.51% (calc. 18.36, 21.77, 9.17, and 36.63), which is interpreted as the loss of C_4_H_6_N_2_O_2_, (SO_4_)_2_+H_2_O, Cl_2_, and C_7_H_7_N_3_O_4_, respectively, from the complexes. The second step occurred at the temperature range of 215°–540°C, which corresponded to the losses of C_11_H_11_Cl_2_NO_2_, C_30_H_32_N_6_O_7_, C_23_H_29_N_6_O_8_, and C_8_H_8_N_2_O_5_ moieties with an estimated weight-loss range of 41.10, 61.11, 18.90, and 39.34% (calc. 41.88, 61.02, 18.36, and 39.42), respectively, from the complexes. The final products were generated over 540, 453, 350, and 515°C with a weight loss of 40.00, 17.12, 25.74, and 24.09% (calc. 39.70, 17.20, 25.99, and 23.88%), and they corresponded to the loss of Zr_2_O_4_, V_2_O_4_, RuO + 7C, and CdO from complexes of Zr(IV), V(IV), Ru(III), and Cd(II), respectively.

In continuation of the thermal investigation, the kinetic and thermodynamic data of complexes (C and D) are listed in Table S1. These data can be summarized in the following:

1. The negative ΔS*∗* indicates that the reactants or intermediates are in a more ordered activated state or have a more rigid structure than the reactants or intermediates, and therefore, the reactions are slower than usual [74]
2. Because ΔH^*∗*^ has positive values, the decomposition processes are endothermic
3. ΔG^*∗*^ has a positive sign, suggesting that the final product's free energy is higher than that of the initial compound and that all degradation steps are nonspontaneous [67]
4. The correlation coefficients of the Arrhenius plots of the thermal decomposition steps were 0.98, showing a good fit with the linear function

### 3.8. Geometric Study

**Figure 5:**
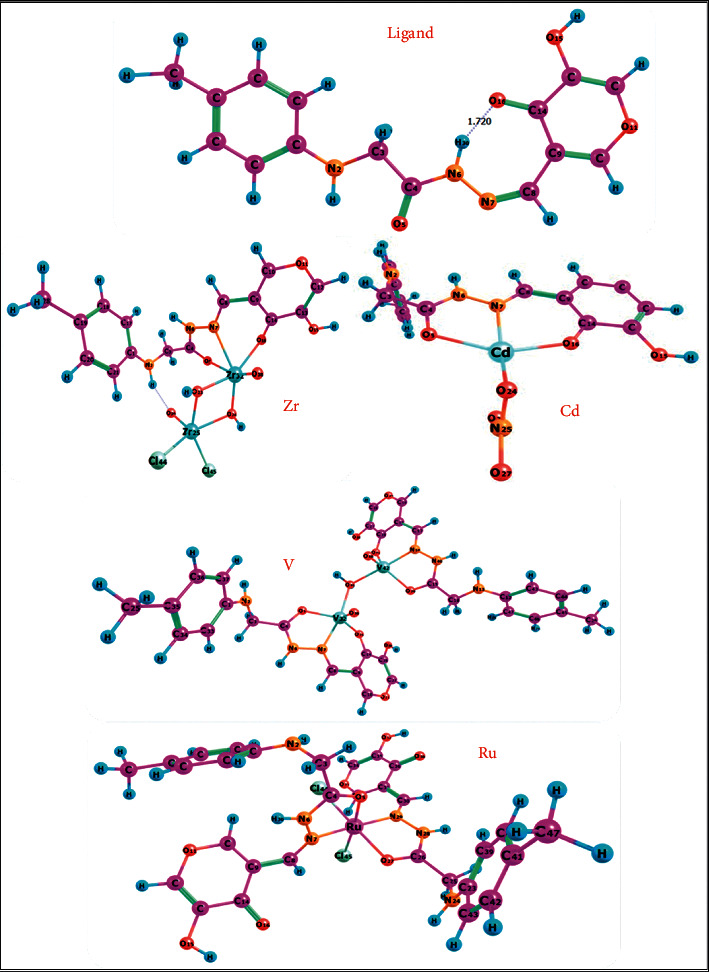
Optimized geometry of the ligand and its metal complexes.

The geometric optimization was carried out for the investigated ligand and its synthesized complexes (Zr(IV), V(IV), Ru(III), and Cd(II)) with the numbered ring system, as seen in Figure 5. The values of optimization energy, dipole moment, energy gap and hardness (*η*), ionization potential (I), electron affinity (A), absolute electronegativity (*χ*), absolute hardness (*η*), and softness (S) are mentioned in Table 6. These molecular properties can be calculated as follows: hardness, *η* = (I − A)/2; softness (S), *S* = 1/2*η*; chemical potential (*μ*), *μ* = −(I + A)/2; and electronegativity (*χ*), *χ* = (I + A)/2. The reactivity of the complexes under the study follows the order V(IV) > Zr(IV) > Cd(II) > Ru(III). As the energy gap of the studied complexes decreases, the reactivity of the complexes increases. The polarity of the ligand increased after complexation by their coordination with Zr(IV) and Cd(II) metal ions and vice versa through its coordination with V(IV) and Ru(III), as indicated by the magnitude of their dipole moments. The lower value of the energy gap is defined as corresponding to a soft molecule, and it explains the charge transfer interactions within the molecule, which influences the molecule's biological activity [65].

**Figure 6:**
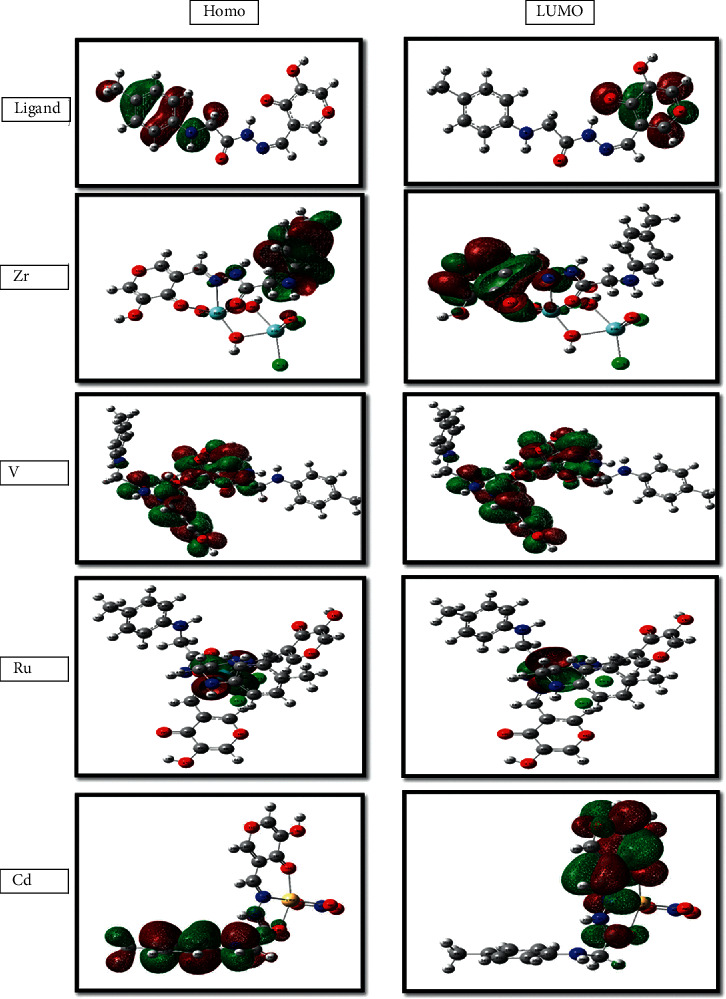
Molecular graphs of the ligand and its metal complexes.

The geometric changes observed in the studied ligand moiety are interesting. Thus, most of the bond lengths were increased upon complexation with different metal ions. Analysis of the data of the bond lengths is presented in Table 7. Thus, we can conclude that the bond lengths of C4-O5, N6-N7, and N7-C8 become longer in all complexes, as the coordination takes place via N atoms of the (C = N) azomethine[Rh] and O5 of the carbonyl group. However, C14-O16 bond length was elongated in all complexes except for the Ru(III) complex because O16 carbonyl group shares in coordination with all-metal ions except for Ru(III). This finding is due to the formation of the M—O and M—N bonds, which make the C—O and C = N bonds weaker [Rh]. The bond angles of the ligands are altered relatively upon coordination, as pointed out in Table 7. The atomic charge distribution of the ligand and its complexes is determined by Mulliken population analysis (MPA). The distribution of positive and negative charges is important from the perspective of an increase or decrease in the bond length between atoms. The results showed that the best negative atomic charges are related to O16 (−0.470), O5 (−0.430), and N7 (−0.115) atoms in the studied ligand. Thus, the metal ions preferred the coordination through O5, N7, and/or O16, forming membered rings. Upon chelation, the charge of the coordinated atoms had a slight decrease in its negative value with the decrease in the remaining surrounding atoms that are relative to the ligands because of their involvement in coordination with the metal ions. The atomic charges in Cd and Ru complexes as representative examples were changed to O16 (−0.402), O5 (−0.421 and −0.294), and N7 (−0.218 and −0.072) atoms, respectively. The electron density on the center of V(IV), Cd(II), Zr(IV), and Ru(III) atoms increased to V(0.757), Cd(1.020), Zr(1.354), and Ru(0.177) after complexation because of the charge transfer from the examined ligand to the central metal ions, i.e., L ⟶ M. Therefore, the theoretical calculations confirmed the results obtained from the analysis tools, which were discussed in the previous characterization part. The generated molecular orbital energy diagrams HOMO and LUMO are presented in Figure 6.

### 3.9. Study on Antibacterial Activity

**Figure 7:**
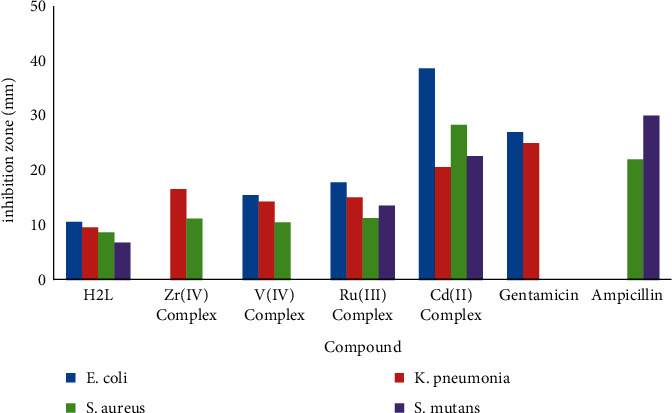
In vitro antibacterial activity of the ligand and its metal complexes.

The antibacterial of the ligand and its complexes (Zr(IV), V(IV), Ru(III), and Cd(II)) were screened against bacterial species such as Gram-negative bacteria (*Escherichia coli* and *Klebsiella pneumonia*) and Gram-positive bacteria (*Staphylococcus aureus* and *Streptococcus mutants*). Ampicillin and gentamicin were used as standard drugs for antibacterial studies. The results of the antibacterial activity of the ligand and complexes are given in Table 8 and Figure 7. These results suggested that the complexes act as a potent antibacterial agent more than the ligand due to their chelation ability. Furthermore, the Cd(II) complex acquired probably the best antibacterial activity followed by the Ru(III) complex towards bacterial species than others, when ampicillin and gentamicin were used as standard drugs.

**Figure 8:**
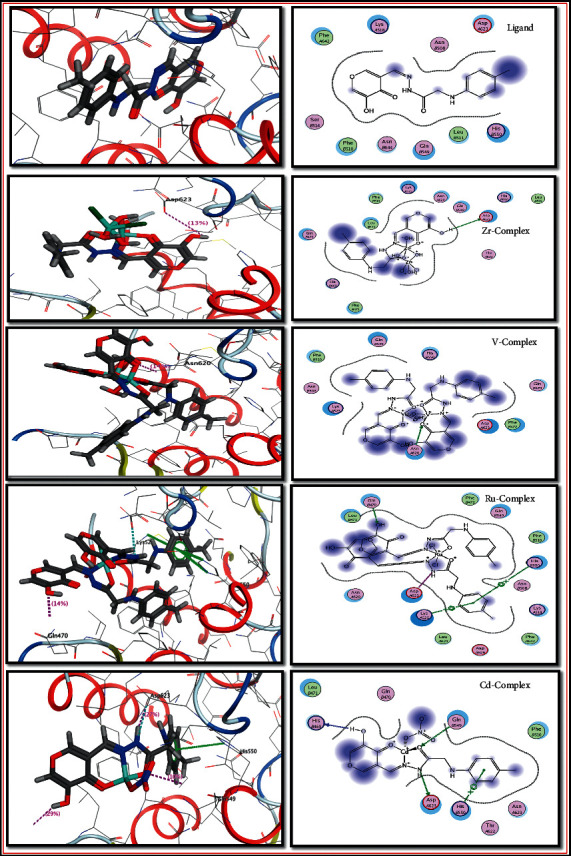
2D and 3D binding affinity of compounds against ribosyltransferase (code: 3GEY).

The docking results revealed interesting interactions between the investigated compounds and the active site amino acids of ribosyltransferase (code: 3GEY). The OH and NH are the most active functional groups that interact with the protein amino acids, as mentioned in Table 9 and Figure 8. According to the scoring energy value, we found the antibacterial activity order of investigated compounds is Ru complex > V complex > Zr complex > Cd complex > ligand.

Experimentally, the inhibition zone values are in good agreement with the previous scoring energy order. But, based on the number of interactions between compounds and the active amino acids of protein, Cd complex gave excellent agreement with the values of zone of inhibition for all studied microorganisms. Thus, we can rearrange the activity sequence to be Cd complex > Ru complex > V complex > ligand > Zr complex. Classically, the polarity of a substance can be specified by its dipole moment property. It has been reported that the drug solubility in water increases with increasing dipole moment; i.e., the dipole moment is an important criterion for deciding the penetration of the drug through the cell membrane of the organism and for the speed of excretion. The lip solubility of the compound increases with decreasing dipole moment, thereby favoring its permeation through the lipid layer of the microorganism more efficiently [75, 76]. Consequently, devastating them more aggressively means that the less-polar drug assists in the penetration of the cell wall and then shifting to the more toxic drug within the cellular environment. The V, Ru, and Cd complexes reflected the lower liposolubility behavior that explains their high antibacterial activities than the ligand and Zr complex.

### 3.10. Cytotoxicity

**Figure 9:**
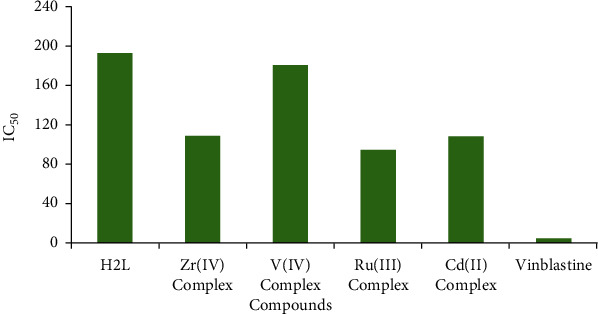
IC_50_ values of the ligand and its complexes against the MCF-7 breast cancer cell line.

The in vitro cytotoxicity of the novel Zr(IV), V(IV), Ru(III), and Cd(II) complexes against the human MCF-7 breast cancer cell line was determined using the MTT assay. The mitochondrial dehydrogenase movement was estimated as a sign of cell viability in terms of optical thickness. The absorbance values were evaluated by nonlinear regression methods to determine the IC_50_ values for the tested compounds in the MCF-7 breast cancer cell line. The cytotoxicity results for the ligand and its complexes of divalent ions Cd(II), trivalent ions Ru(III), and tetravalent ions against the MCF-7 breast cancer cell line at concentrations of 31.25, 62.5, 125, 250, 500, and 1000 *μ*g/ml based on the surviving fraction results and IC_50_ values for the different compounds are shown in Figure 9.

Among the tested compounds, Ru(III) complex (IC_50_ = 94.37 *μ*g/ml) exhibited the greatest activity against the MCF-7 breast cancer cell line. The IC_50_ values for Cd(II), Zr(IV), and V(IV) complexes and the ligand are in the range of 107.97, 108.5, 180.19, and 192.37 *μ*g/ml, respectively, and they were comparable with that of vinblastine (4.58 *μ*M) [77].

The IC_50_ values followed the order vinblastine < Ru(III)L < Cd(II)L < Zr(IV)L < V(IV)L < L. From the obtained results, it is obvious that the prepared ligand and its complexes are potent agents against the MCF-7 breast cancer cell lines. Importantly, the Ru(III) complex, in particular, was more potent than the other complexes and showed concentration-dependent effects, which could suggest its potential use in cancer therapy.

**Figure 10:**
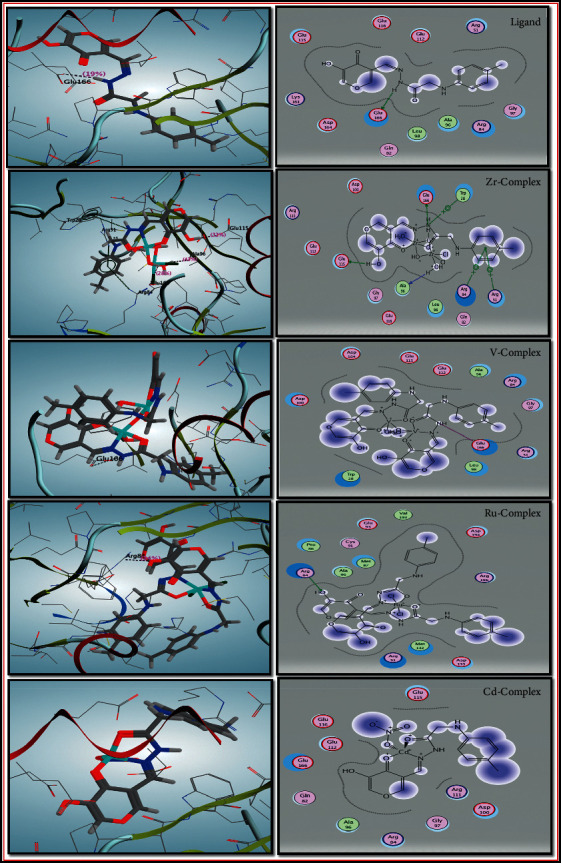
2D and 3D compound interactions with the active site of the NUDT5.

It is known that NUDT5 (nucleotide diphosphate hydrolase type 5) is an upstream regulator of tumor drivers and a biomarker for cancer stratification, as well as a target for drug discovery towards the treatment of aggressive cancer types and metastasis [78]. The enzyme NUDT5 catalyzes the ADP (adenosine diphosphate) ribose derived from hydrolysis of poly(ADP-ribose), and pyrophosphate (PPi) is converted to ATP. Thus, NUDT5 is an attractive target for drug design against breast cancer. The amino acid residues involved in the binding pockets with synthesized molecules are thus predicted. Six possible binding residues mentioned in Table 10 and Figure 10 were involved in interaction with our compound inhibitors. The theoretical sequence interaction activity was compatible with all compounds except for the Ru complex. It may be due to the mechanism of its action being more effective with other types of significant target proteins that control the breast tumor progression.

## 4. Conclusions

Four novel complexes of Zr(IV), V(IV), Ru(III), and Cd(II) based on ligand N'-((5-hydroxy-4-oxo-4H-pyran-3-yl)methylene)-2-(p-tolylamino)acetohydrazide(H_2_L) were isolated. The structures of the ligand and its complexes were proposed based on the results of different characterization techniques, which confirm the formation of 2 : 1 of Zr(IV), 2 : 2 V(IV), 1 : 2 of Ru(III), and 1 : 1 (M : L) of Cd(II) complexes. The nonelectrolytic nature of the chelates was observed by their molar conductance values, except for the V(IV) and Cd(II) complexes, which are ionic. The thermal stability of the ligand and complexes was also confirmed. The antibacterial efficacy was tested against a variety of bacteria strains and compared to the well-known standard drugs, ampicillin and gentamicin. These results suggested that complexes act as a potent antibacterial agent than the ligand due to their chelation ability. Furthermore, the Cd(II) complex acquired better antibacterial activity, followed by the Ru(III) complex towards bacterial species than others. Moreover, the prepared ligand and its complexes are potent agents against the MCF-7 breast cancer cell lines. The IC_50_ values followed the order Vinblastine < Ru(III)L < Cd(II)L < Zr(IV)L < V(IV)L < L. DFT calculation was carried out for the prepared compounds. The synthesized ligand and its all-metal complexes had a satisfactory spectrum for their antibacterial activity against bacterial species. The docking simulation pointed out the binding model of all compounds. The interactions with active site amino acids of ribosyltransferase (code: 3GEY) and NUDT5 (PDB code : 5NWH) of interest in treating bacterial infection and breast cancer, respectively, involved the hydrogen bond formation and arene-cation interaction. Therefore, the current findings provide a new chance for the development and discovery of antimicrobials to beat the ever-increasing drug resistance problem.

## Figures and Tables

**Table 1:** Physicochemical parameters of the ligand and its complexes of Zr(IV), V(IV), Ru(III), and Cd(II) ions.

Compounds molecular formula	M. Wt.	Color yield (%)	Conductivity (Ω^−1^·cm^2^·mol^−1^)	(Cal.) found (%)			
				C	H	N	M
C_15_H_15_N_3_O_4_	301.30	Yellow 94	—	59.80 (59.76)	5.02 (4.98)	13.95 (13.92)	—
C_15_H_17_Cl_2_N_3_O_8_Zr_2_	620.66	Yellow 88	46	29.03 (28.97)	2.76 (2.71)	6.77 (6.72)	29.40 (29.33)
C_30_H_34_N_6_O_20_S_2_V_2_	964.63	Yellow 83	89	37.35 (37.12)	3.55 (3.49)	8.71 (7.68)	10.56 (10.52)
C_30_H_29_Cl_2_N_6_O_8_Ru	773.57	Deep brown 86	47	46.58 (46.53)	3.78 (3.74)	10.86 (11.82)	13.07 (13.02)
C_15_H_15_CdN_5_O_10_	537.72	Yellow 89	71	33.51 (33.24)	2.81 (2.76)	13.02 (12.98)	20.91 (20.89)

**Table 2:** FT-IR spectral bands of the ligand and its complexes of Zr(IV), V(IV), Ru(III), and Cd(II) in 4000–400 cm^−1^.

Compound	*ν* (OH/H_2_O)	*ν* (N–H)	*ν* (C = O)_side_	*ν* (C = O)_ring_	*ν* (C = N)	*ν* (M-O)	*ν* (M-N)
C_15_H_15_N_3_O_4_	3392	3202	1677	1639	1604	—	—
C_15_H_17_Cl_2_N_3_O_8_Zr_2_	3434	2625	1692	1644	1595	621	553
C_30_H_34_N_6_O_20_S_2_V_2_	3433	2925	1689	1650	1553	605	560
C_30_H_29_Cl_2_N_6_O_8_Ru	3399	3100	1691	1644	1582	571	506
C_15_H_15_CdN_5_O_10_	3422,3327	3234,3104	1669	1638	1603	587	455

**Table 3:** The electronic spectra and magnetic data of the ligand and its Zr(IV), V(IV), Ru(III), and Cd(II) complexes.

Compound	Wavelength *λ*_max_ (nm)	Assignment	*μ*_eff_ (BM)
C_15_H_15_N_3_O_4_	341 399	n-*π*^*∗*^ n-*π*^*∗*^	—

C_15_H_17_Cl_2_N_3_O_8_Zr_2_	338 479	n-*π*^*∗*^ d–d	—

C_30_H_34_N_6_O_20_S_2_V_2_	351 555	n-*π*^*∗*^ d–d	1.6

C_30_H_29_Cl_2_N_6_O_8_Ru	340 519	n-*π*^*∗*^ d–d	1.74

C_15_H_15_CdN_5_O_10_	338 378	n-*π*^*∗*^ n-*π*^*∗*^	Dia

**Table 4:** PXRD data of the ligand and Zr(IV), Ru(III), and Cd(II) complexes.

Compound	Angle in 2*θ*	*d* value in nm	FWHM	Grain size in nm
C_15_H_15_N_3_O_4_	15.822	0.559119	0.238	37.48
C_15_H_17_Cl_2_N_3_O_8_Zr_2_	12.407	0.700383	1.915	4.64
C_30_H_29_Cl_2_N_6_O_8_Ru	13.092	0.657801	2.558	3.47
C_15_H_15_CdN_5_O_10_	26.855	0.331720	0.214	42.42

**Table 5:** Thermal data (TGA\DTG) of the ligand and its Zr(IV), V(IV), Ru(III), and Cd(II) metal complexes.

Compound	Temp. range/°C DTG	Temp. range/°C TGA	Wt. loss (%) Calc. (F.)	Assignments
C_15_H_15_N_3_O_4_ residue	195, 456	197–391 >391	60.11 (60.31) 39.88 (39.68)	C_7_H_5_N_2_O_4_ complete decomp.

C_15_H_17_Cl_2_N_3_O_8_Zr_2_ residue	30, 340, 548	20–300 300–540 >540	18.36 (18.90) 41.88 (41.10) 39.70 (40.00)	C_4_H_6_N_2_O_2_ C_11_H_11_Cl_2_NO_2_ Zr_2_O_4_

C_30_H_34_N_6_O_20_S_2_V_2_ residue	295, 380, 445	20–248 248–453 >453	21.77 (21.73) 61.02 (61.11) 17.20 (17.12)	(SO_4_)_2_+H_2_O C_30_H_32_N_6_O_7_ V_2_O_4_

C_30_H_29_Cl_2_N_6_O_8_Ru residue	285, 743	25–215 215–350 >350	9.17 (8.98) 18.36 (18.90) 25.99 (25.74)	Cl_2_ C_23_H_29_N_6_O_8_ RuO+7C

C_15_H_15_CdN_5_O_10_ residue	238, 257, 377	176–310 332–515 >515	36.63 (36.51) 39.42 (39.34) 23.88 (24.09)	C_7_H_7_N_3_O_4_ C_8_H_8_N_2_O_5_ CdO

**Table 6:** Ground-state properties of the ligand and its metal complexes by using B3LYP/6-311G and B3LYP/LANL2DZ, respectively.

Parameter	Ligand	Zr complex	V complex	Ru complex	Cd complex
E_T_, Hartree	−1045.39976321	−1471.25957091	−2460.74826616	−2215.06501660	−1373.95124556
E_HOMO_, eV	−4.64634638	−5.43139524	−2.28167581	−5.84500851	−6.04474017
E_LUMO_, eV	−2.41038572	−3.87490322	−1.91459403	−1.69363747	−2.21391942
ΔE, eV	2.23596066	1.556492	0.367082	4.151371	3.830821
I = -E_HOMO_, eV	4.64634638	5.43139524	2.28167581	5.84500851	6.04474017
A = -E_LUMO_, eV	2.41038572	3.87490322	1.91459403	1.69363747	2.21391942
*χ*, eV	3.156018004	5.979020991	11.43143046	1.815941266	2.155846
*η*, eV	1.11798033	0.77824601	0.18354089	2.07568552	1.915410375
S, eV^−1^	0.44723506	0.642470367	2.724188599	0.240884274	0.261040666
*μ*, eV	−3.52836605	−4.65314923	−2.09813492	−3.76932299	−4.129329795
Dipole moment (Debye)	10.5480	24.5000	6.6523	7.1655	12.4971

**Table 7:** The optimized bond lengths, Å, and bond angles, degrees, for the ligand and complexes by using B3LYP/6-311G and B3LYP/LANL2DZ, respectively.

Bond length (A°)	Ligand	Zr complex	V complex	Ru complex	Cd complex

R (C3-C4)	1.52898	1.53025	1.53629	1.52582	1.52634
R (C4-O5)	1.24188	1.26253	1.27911	1.27476	1.28799
R (C4-N6)	1.38249	1.39983	1.37457	1.37074	1.34966
R (N6-N7)	1.36156	1.38935	1.37457	1.41239	1.40204
R (N7-C8)	1.29829	1.30082	1.30634	1.30675	1.36844
R (C8-C9)	1.48124	1.46948	1.46238	1.47716	1.42294
R (C9-C14)	1.47007	1.47220	1.45316	1.46690	1.46884
R (C14-O16)	1.26148	1.27806	1.30469	1.26956	1.28209
R (H30-N6)	1.03165	1.02382	1.01981	1.01621	1.02071
R (O16-M)	---	2.30401	1.97868	---	2.22725
R (N7-M)	---	2.45138	2.04808	2.04956	2.27958
R (O5-M)	---	2.46681	2.05132	2.12299	2.29550
R (O24-M)	---	---	---	---	2.30836
R (Cl-M)	---	2.50060	---	2.47839	---
R (O38-M32)	---	1.81411	1.61500	---	---
R (O49-M32)	---	---	1.61500	---	---
R (O49-V42)	---	---	2.24487	---	---
R (O23-Zr32)	---	2.09705	---	---	---
R (O24-Zr32)	---	2.10093	---	---	---
R (O23-Zr25)	---	2.25072	---	---	---
R (O24-Zr25)	---	2.28531	---	---	---

*Bond angles, degrees*					

A (O16-C14-C9)	124.823	123.549	123.571	125.425	123.971
A (C14-C9-C8)	127.301	120.637	122.951	117.688	123.121
A (C9-C8-N7)	136.152	119.512	121.269	128.038	125.348
A (C8-N7-N6)	123.820	122.388	119.179	121.058	115.514
A (N7-N6-C4)	119.717	114.193	112.913	117.042	118.976
A (N6-C4-O5)	125.039	118.233	117.812	119.976	121.413
A (N6-C4-C3)	112.721	117.986	118.781	118.408	117.475
A (C4-C3-N2)	108.710	108.786	108.342	108.607	113.703
A (H30-N6-N7)	118.934	122.793	119.034	120.689	122.445
A (O16-M-N7)	---	69.500	86.972	---	77.842
A (O5-M-N7)	---	63.039	76.570	79.073	72.433
A (O5-M-O24)	---	---	---	---	102.185
A (C14-O16-M)	---	117.963	126.871	---	137.572
A (-C8-N7-M)	---	124.857	127.103	128.034	132.095
A (Cl45-M-Cl44)	---	106.169	---	96.098	---
A (Cl45-Ru-O5)	---	---	---	174.603	---
A (O16-V32-O38)	---	---	113.912	---	---
A (V32-O49-V42)	---	---	120.827	---	---
A (Zr32-O23-Zr25)	---	106.677	---	---	---

**Table 8:** Results of antibacterial bioassay of the ligand (H2L) and its complexes of Zr(IV), V(IV), Ru(III), and Cd(II) against different strains of bacteria.

Microorganism	Sample					
	H2L	Zr (IV) complex	V (IV) complex	Ru (III) complex	Cd (II) complex	Standard antibiotic
Gram-negative bacteria	Gentamicin					
*Escherichia coli* (ATCC:10536)	10.6 ± 0.5	0	15.5 ± 0.6	17.8 ± 0.6	38.6 ± 0.6	27 ± 0.5
*Klebsiella pneumonia* (ATCC:10031)	9.6 ± 0.6	16.6 ± 0.6	14.3 ± 0.5	15.1 ± 0.5	20.6 ± 0.6	25 ± 0.5

Gram-positive bacteria	Ampicillin					
*Staphylococcus aureus* (ATCC:13565)	8.7 ± 0.6	11.2 ± 0.5	10.5 ± 0.5	11.3 ± 0.5	28.3 ± 0.6	22 ± 0.1
*Streptococcus mutans* (ATCC:25175)	6.8 ± 0.5	0	0	13.6 ± 0.5	22.6 ± 0.6	30 ± 0.5

**Table 9:** Binding affinity of compounds against ribosyltransferase (code: 3GEY).

Docking 3GEY			
Compound	Scoring energy (RMSD)	Involved amino acids	Type of interaction
Ligand	−4.5209 (1.9)	---	Solvent contact
Zr(IV) complex	−7.2857 (0.8)	Asp-*A*623	Side chain acceptor
V(IV) complex	−8.4417 (2.6)	Asn-*A*620	Side chain donor
Ru(III) complex	−8.4842 (2.8)	(His-*B*550 and Lys-*A*525) and Gln-*B*470	Arene-cation interaction and side chain donor
Cd(II) complex	−5.5194 (2.9)	Gln-*B*549, Asp-*A*623, His-*B*469 and His-*B*550	Side chain donor, side chain acceptor, backbone acceptor, and arene-cation interaction

**Table 10:** Binding affinity of compounds against the breast cancer regulator NUDT5 (PDB code : 5NWH).

Docking: 5NWH			
Compound	Scoring energy (RMSD)	Involved amino acids	Type of interaction
Ligand	−3.7471 (1.0)	Glu-166	Side chain acceptor
Zr (IV) complex	−9.2570 (2.7)	Glu-166, Glu-115, Ala-96, and (Arg-84, Arg-51, and Trp-28)	Side chain acceptor, backbone acceptor, and arene-cation interaction
V (IV) complex	−6.6536 (3.0)	Glu-*166*	Metal contact
Ru (III) complex	−3.2467 (2.9)	Arg-84	Side chain donor
Cd (II) complex	−10.2435 (2.5)	---	Solvent contact

## Data Availability

Data will be made available on request.

## Supplementary Materials

Supplementary Materials Table S1 : kinetic and thermodynamic data of complexes (C and D). Figure S1 : ^1^H-NMR (A) and ^13^C-NMR (B) spectrum of the ligand in DMSO-d6. Figure S2 : FT-IR spectra of (A) Zr(IV), (B) V(IV), and (C) Ru(III) complexes. Figure S3 : the mass spectra of the ligand and Zr(IV) and V(IV)complexes. Figure S4 : PXRD powder pattern of Zr(IV) and V(IV) complexes. Figure S5 : PXRD powder pattern of the ligand and Cd(II) complex. Scheme S1 : mass fragmentation of the ligand and Zr(IV) and V(IV) complexes.
