# Supplementary material for: Synthesis, Characterization, PXRD Studies, Theoretical Calculation, and Antitumor Potency Studies of a Novel N,O-Multidentate Chelating Ligand and Its Zr(IV), V(IV), Ru(III), and Cd(II) Complexes
Source: Bioinorg Chem Appl. 2022 Jun 28;2022:2006451. doi: 10.1155/2022/2006451 (PMC10908574; doi:10.1155/2022/2006451)
Supplement: Supplementary Materials — Table S1 : kinetic and thermodynamic data of complexes (C and D). Figure S1 : 1H-NMR (A) and 13C-NMR (B) spectrum of the ligand in DMSO-d6. Figure S2 : FT-IR spectra of (A) Zr(IV), (B) V(IV), and (C) Ru(III) complexes. Figure S3 : the mass spectra of the ligand and Zr(IV) and V(IV)complexes. Figure S4 : PXRD powder pattern of Zr(IV) and V(IV) complexes. Figure S5 : PXRD powder pattern of the ligand and Cd(II) complex. Scheme S1 : mass fragmentation of the ligand and Zr(IV) and V(IV) complexes. [file 2006451.f1.docx]

| **No.** | **Complex** | **E*** (kJ mol^-1^) | **A** (S^-1^) | **∆S*(**J mol^-1^ K^-1^**)** | **∆H*(**kJ mol^-1^**)** | **∆G*(**kJ mol^-1^**)** | ^a^R^2^ |
| --- | --- | --- | --- | --- | --- | --- | --- |
| **C** | C_30_H_29_Cl_2_N_6_O_8_Ru | 41.61 | 3.92 E^9^ | −8.15 | 36.24 | 41.5 | 0.98 |
| **D** | C_15_H_15_CdN_5_O_10_ | 29.27 | 5.80 E^9^ | -7.76 | 23.91 | 28.91 | 0.98 |

**Table S1** : Kinetic and thermodynamic data of complexes (**C and D**).

^a^ R^2^: Correlation coefficient.

| 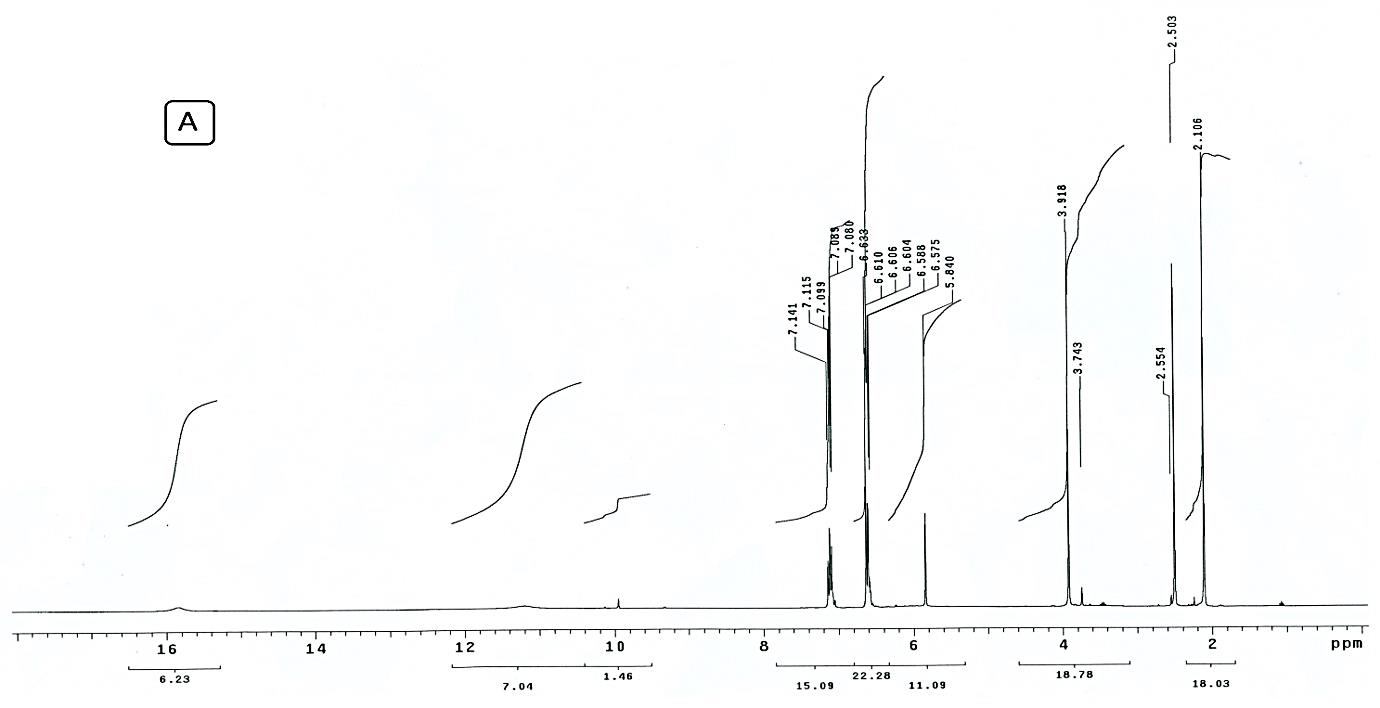  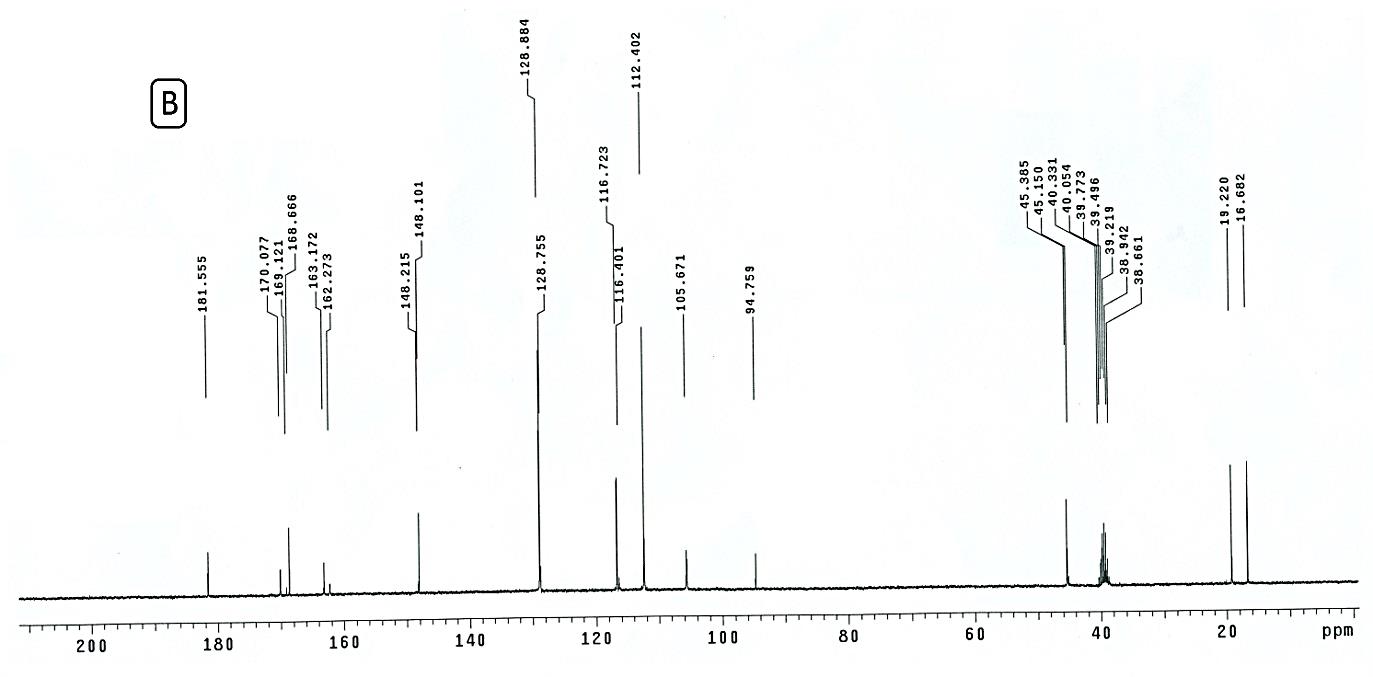 |
| --- |

**Figure S1.** ^1^H-NMR (A) and ^13^C-NMR (B) spectrum of the ligand in DMSO d6


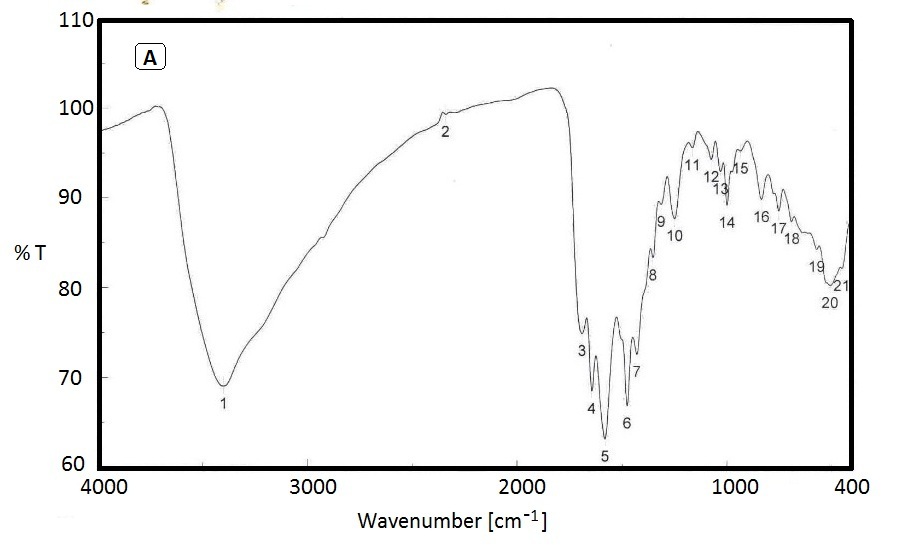

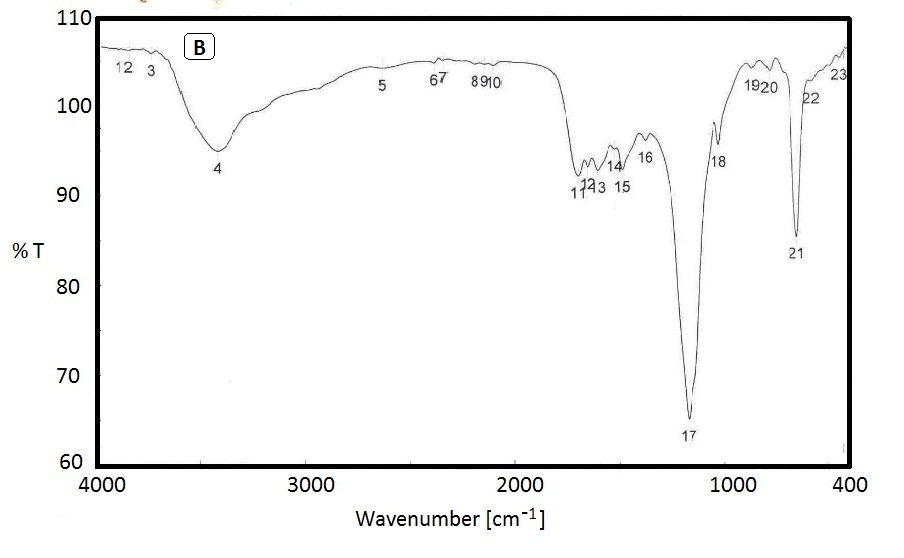

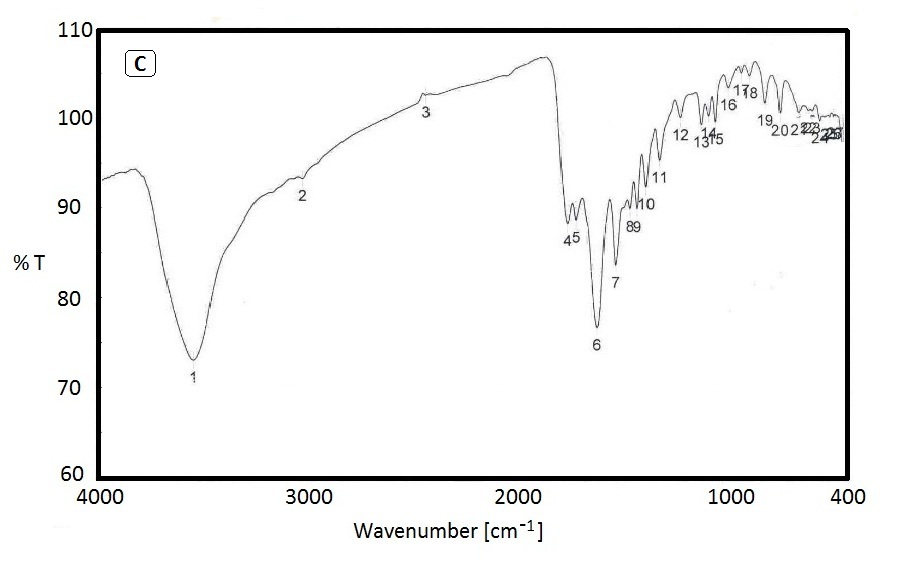


**Figures S2.** FT-IR spectra of (A) Zr(IV), (B) V(IV) and (C) Ru(III) Complexes

|  |
| --- |

**Figure S3**: The mass spectra of Ligand, Zr(IV) and V(IV)complexes

**Scheme S1:** Mass fragmentation of Ligand, Zr(IV) and V(IV)complexes

|  |
| --- |

**Figure S4**: PXRD powder pattern of Zr(IV) and Ru(III) complexes.

**Figure S5**: PXRD powder pattern of ligand and Cd (II) complex.
